# The Overexpression of *ORR3* Negatively Regulates the Growth of Young Rice Roots by Reducing the Cell Size and the Number in the Root Meristematic Zone

**DOI:** 10.3390/plants14111627

**Published:** 2025-05-27

**Authors:** Gang Wei, Wenjing Yu, Xinlong Chen, Han Yun, Tongming Wang, Nan Wang, Ting Zhang, Guanghua He

**Affiliations:** 1 Chongqing Key Laboratory of Crop Molecular Improvement, Rice Research Institute, Academy of Agricultural Sciences, College of Agronomy and Biotechnology, Southwest University, Chongqing 400715, China; weilj95@163.com (G.W.); yuwenjing064@163.com (W.Y.); 15225371326@163.com (H.Y.); wtming@hotmail.com (T.W.); wangnan_xndx@126.com (N.W.); 2 Integrative Science Center of Germplasm Creation in Western China (Chongqing) Science City, College of Agronomy and Biotechnology, Southwest University, Chongqing 400715, China; slndchenxinlong@163.com

**Keywords:** B-type cytokinin response regulator factor, root, cell wall, auxin, rice (*Oryza sativa* L.)

## Abstract

The growth of young roots is crucial for the development and yield of rice. However, the molecular mechanisms underlying young rice root development remain unclear. Our research indicates that the rice B-type cytokinin response regulator factor *ORR3* negatively regulates the development of young rice roots. *ORR3* is highly expressed in the root meristematic zone of young rice roots. In transgenic lines overexpressing *ORR3*, the lengths of primary roots and adventitious roots, as well as the corresponding root meristematic zone lengths, are significantly reduced. This is due to a decrease in both the number and size of longitudinal cells in the root meristematic zone. On the one hand, *ORR3* can inhibit root apical cell division and reduce the number of longitudinal cells in the root meristematic zone by affecting the auxin synthesis and transport pathways. On the other hand, ORR3 may directly activate the transcription of cell wall metabolism-related genes, thereby restricting the size of cells in the root meristematic zone. In summary, *ORR3* negatively regulates rice young root growth by responding to cytokinin signals to influence auxin signal transduction and cell wall metabolism pathways, thereby negatively regulating the number and size of cells in the root meristematic zone.

## 1. Introduction

Roots are emerging as a key target for the second green revolution and are considered crucial for the production of high-yield crops such as wheat and rice [1]. Therefore, it is urgent to decipher the molecular regulatory networks in the root tips of cereal crops. The roots of young plants mainly consist of primary roots and adventitious roots. After seed germination, the primary root first breaks through the seed coat and grows, followed by the emergence of adventitious roots from the base of the stem or the tillering node. In cereal crops, the growth of the primary root is the foundation for seedling establishment and grain yield [2]. Adventitious roots, on the other hand, are the main components of the root system in cereals and play a vital role in nutrient uptake, stress resistance, and yield [3]. Unlike the continuously growing primary roots in dicotyledonous plants [4,5], the primary roots of monocotyledonous plants such as rice exhibit determinate growth. These primary roots grow rapidly for a short period (about 7–10 days) after germination, and then cease to grow [6]. As a result, adventitious roots are the main components of the root system in monocotyledonous plants like rice, providing support throughout their life cycle [3,7]. Despite the differences in root structure and function between monocotyledonous and dicotyledonous plants, they share many conserved mechanisms in the formation of root radial patterns. These include the gene regulatory networks, cell division and differentiation, and hormone regulation. This suggests that plants have retained certain key developmental regulatory pathways during evolution to adapt to different growth environments [8,9,10].

The internal factors influencing root development mainly include hormonal regulation, cell wall metabolism, and gene regulation, among others. Multiple plant hormones have been shown to participate in the regulation of root development [11], such as cytokinins [12,13] and auxins [14]. For instance, the cytokinin efflux transporter ABCC4 regulates the number of cells in the root meristem by modulating the cytokinin levels, thereby controlling the growth of the primary root in *Arabidopsis thaliana* [15]. In rice, *OsMADS25* regulates root system development through auxin signaling based on the Aux/IAA pathway [16]. The overexpression of the wheat gene *TaARF4* in *Arabidopsis* leads to elevated levels of free IAA, which inhibits apical dominance and reduces the primary root length [17]. Hormones can interact with each other to regulate root development in either a synergistic or antagonistic manner. Cytokinins (CTKs) and auxins (IAAs) exhibit antagonistic effects during root formation [18]. A high endogenous IAA/CTK ratio is conducive to adventitious root formation, while a low ratio is unfavorable for root induction [19].

Plant root development is also closely related to cell wall biosynthesis [20]. The *AtCSLD3* gene, a member of the cellulose synthase-like (subfamily D) gene family in *Arabidopsis thaliana*, plays a role in root hair growth [21]. The *OsGLU3* gene encodes an endo-1,4-*β*-glucanase that regulates cell wall relaxation, which is essential for rice root elongation [22,23]. Cell wall metabolism can also influence plant root development by regulating hormones. The overexpression of *AtUGT84B1* leads to auxin deficiency in the root system of Arabidopsis, resulting in significant root growth defects [24]. In rice, the overexpression of the Arabidopsis gene *AtUGT76C2* reduces the cytokinin (CTK) levels and promotes root system growth [25]. Gene regulation is equally crucial for plant root development. The WUSCHEL-related homeobox transcription factor OsWOX4 in rice may directly activate the target gene *OsAUX1*, thereby affecting auxin accumulation and regulating primary root development [9]. OsFPFL4 controls auxin accumulation by influencing auxin biosynthesis and transport, and the overexpression of *OsFPFL4* leads to increased auxin accumulation in the rice root system, ultimately resulting in shorter primary roots [26].

Xyloglucan endotransglycosylase/hydrolase (XTH) is involved in cell wall remodeling and has a significant impact on root elongation and lateral root development. Some studies on transgenic *Arabidopsis thaliana* overexpressing pepper *CaXTH3* have shown that CaXTH3 may participate in cell wall remodeling to strengthen the cell wall layers [27]. The conversion between methyl-esterified and de-methyl-esterified pectin determines the degree of cross-linking in the cell wall, which is crucial for cell morphogenesis [28,29]. Cell wall defects often induce changes in plant hormone signaling. For example, perturbations in pectin modification and xyloglucan production can weaken the auxin responses [30]. Auxin can trigger genome-wide transcriptional programming in *Chara braunii*, upregulating the pectin lyase PLL gene involved in cell wall remodeling and participating in the regulation of cell expansion [31].

In rice, seven B-type cytokinin response regulator factors (RRs) have been reported. These genes encode proteins with conserved receptor domains, GARP DNA-binding domains, and non-conserved C-terminal regions. The sequence of the C-terminal region may determine the distinct transcriptional activities of ORRs proteins [32]. Cytokinin signaling is a phosphorelay process. The receptor kinase AHK, activated by cytokinin signaling, phosphorylates AHP (Arabidopsis homologs of Histidine-containing Phosphotransmitter) proteins. These phosphorylated AHP proteins shuttle between the cytoplasm and the nucleus, transferring phosphate groups to the receptor domains of B-type ARR (Arabidopsis Response Regulator) proteins. The activated B-type ARRs function as transcription factors to activate or repress the expression of downstream genes [32,33,34]. However, the mechanisms by which B-type cytokinin response factors regulate root growth in plants remain unclear. In this study, we found that *ORR3* may negatively regulate the number and size of cells in the root meristematic zone of rice by influencing hormone pathways and cell wall metabolism, thereby restricting the development of young rice roots. Overall, the functional study of *ORR3* enriches our understanding of rice root system development.

## 2. Result

### 2.1. Expression Pattern and Homology Analysis of ORR3, and Subcellular Localization of ORR3 Protein

**Figure 1:**
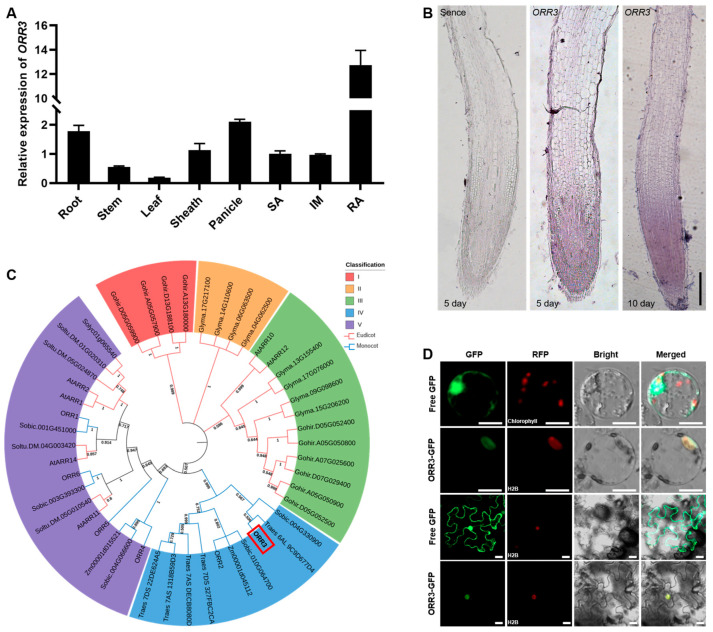
The expression pattern, phylogenetic tree, and subcellular localization of ORR3. (**A**) The expression levels of *ORR3* were analyzed in the root, stem, leaf, sheath, panicle, shoot apical SA (SA), inflorescence meristem (IM), and root apical (RA) using qRT-PCR, with *ACTIN* serving as the internal control. (**B**) The in situ hybridization of *ORR3* in the root apical of the primary root (5-day-old) and the adventitious roots (10-day-old). Scale bar = 0.2 mm. (**C**) The phylogenetic analysis of ORR3 in monocotyledons and dicotyledons; the red box is marked ORR3. (**D**) The co-localization of ORR3-GFP with H2B-RFP (H2B, as one of the core histones of the nucleosome, can serve as a nuclear marker) in rice protoplasts and *Nicotiana benthamiana* leaves. Scale bar = 20 μm.

To elucidate the role of *ORR3* in rice growth and development, we analyzed its expression in various rice tissues using qRT-PCR. The results showed that *ORR3* is expressed in all tissues, but notably, its expression level is higher in roots and panicles, especially in the root apical, where the expression level is extremely high (Figure 1A). This suggests that *ORR3* may primarily play a role in rice root development and may also contribute to panicle development. Subsequently, we used mRNA in situ hybridization to analyze the expression of *ORR3* in the root tips and found that it is mainly expressed in the root meristematic zone (Figure 1B), indicating that *ORR3* may be involved in regulating the development of the rice root meristematic zone. To investigate the homology of ORR3, we constructed a phylogenetic tree using the amino acid sequence of ORR3, including species from both monocots and dicots (Figure 1C and Figure S1B). The results suggest that type-B cytokinin response factors may be evolutionarily conserved between the monocots and the dicots, implying that their functions may be similar across different plants. Finally, we generated an ORR3-GFP fusion protein and analyzed its subcellular localization in rice protoplasts and tobacco mesophyll cells. The results showed that ORR3 is localized in the nucleus (Figure 1D).

### 2.2. The Overexpression of ORR3 Leads to a Reduction in Both the Number and Size of Cells in the Root Meristematic Zone of Rice Seedlings

**Figure 2:**
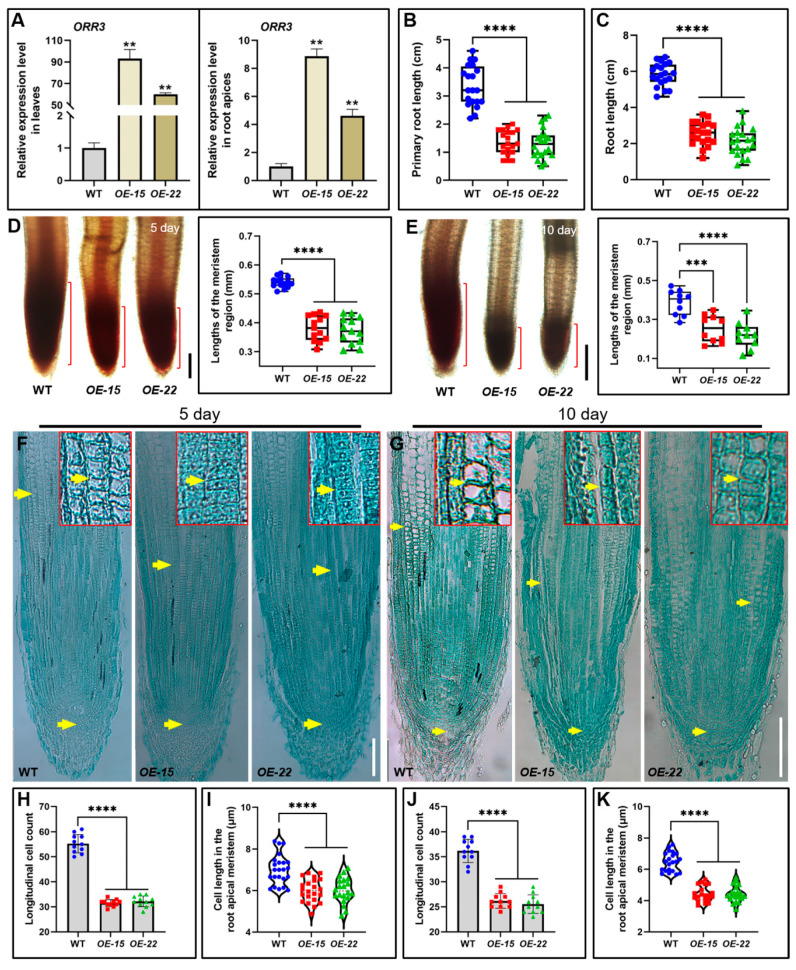
Phenotypic analysis of *ORR3-OE* lines. (**A**) Expression of *ORR3* in *ORR3-OE* lines. (**B**) Statistics of root length of 5-day-old seedlings, *n* = 20. (**C**) Statistics of root length of 10-day-old seedlings, *n* = 20. (**D**) Acetic carmine staining of root meristematic zone (red bracket) and length measurement of 5-day-old seedlings. Scale bar = 0.2 mm, *n* ≥ 13. (**E**) Acetic carmine staining of root meristematic zone (red bracket) and length measurement of 10-day-old seedlings. Scale bar = 0.2 mm, *n* = 10. (**F**) Longitudinal paraffin sections of root tips of primary roots of 5-day-old seedlings (yellow arrow indicates length of root meristematic zone; red box: partially enlarged in size). Scale bar = 0.1 mm. (**G**) Longitudinal paraffin sections of adventitious root tips of 10-day-old seedlings (yellow arrow indicates length of the root meristematic zone; red box: partially enlarged in size). Scale bar = 0.1 mm. (**H**) Number of longitudinal cells in root tips of primary roots of 5-day-old seedlings, *n* = 11. (**I**) Longitudinal cell length of root tip of primary roots of 5-day-old seedlings, *n* ≥ 20. (**J**) Number of longitudinal cells in root tips of adventitious roots of 10-day-old seedlings, *n* = 11. (**K**) Length of root tip cells of adventitious roots of 10-day-old seedlings, *n* ≥ 20.** *p* < 0.01, *** *p* < 0.001, **** *p* < 0.0001, determined by Student’s *t*-test.

To further explore the role of *ORR3* in rice development, we generated overexpression lines of *ORR3* and identified two effective transgenic lines, *OE-15* and *OE-22*, through qRT-PCR validation (Figure 2A). Phenotypic analyses were then conducted on 5-day-old and 10-day-old seedlings of WT and *ORR3-OE* transgenic lines. The results showed that the primary roots of the 5-day-old *OE-15* and *OE-22* seedlings were significantly shorter than those of the WT, with reductions of about 60% and 61%, respectively (Figure 2B). Additionally, plant height was also significantly reduced in these lines (Figure S2A,B). Conversely, while the adventitious roots of the 10-day-old *OE-15* and *OE-22* seedlings were significantly shorter than those of the WT, with reductions of about 56% and 64%, respectively (Figure 2C), plant height in these lines increased significantly (Figure S2C,D). These observations indicate that the overexpression of *ORR3* negatively regulates the root development of young rice.

To elucidate the cause of the shortened roots in the *ORR3-OE* transgenic seedlings, we employed aceto-carmine staining, which highlights actively dividing cells in the root meristematic zone [35]. The staining results revealed that the length of the meristematic zone in the primary roots of the 5-day-old *OE-15* and *OE-22* seedlings was significantly reduced compared to that of the WT, with decreases of about 29% and 31%, respectively (Figure 2D). Similarly, the length of the meristematic zone in the adventitious roots of the 10-day-old *OE-15* and *OE-22* seedlings was significantly reduced compared to that of the WT, with decreases of about 35% and 44%, respectively (Figure 2E). Further analysis using the paraffin sectioning of the root tips at 5 days and 10 days showed that the meristematic zone length in *OE-15* and *OE-22* was markedly shorter than that in the WT (Figure 2F,G). The cell size measurements indicated that the cell length in the meristematic zone of *OE-15* and *OE-22* was significantly reduced, and the number of longitudinal cells was significantly decreased (Figure 2H–K). These findings suggest that the short-root phenotype in the *ORR3-OE* lines is attributed to the reduced length and number of cells in the meristematic zone.

In summary, the overexpression of *ORR3* significantly decreased the length of the primary and adventitious roots in rice. This phenotype is primarily caused by the reduced length and number of cells in the root meristematic zone, likely due to *ORR3* overexpression inhibiting cell division in this region.

### 2.3. ORR3 Inhibits Root Tip Cell Division by Affecting the Auxin Pathway

**Figure 3:**
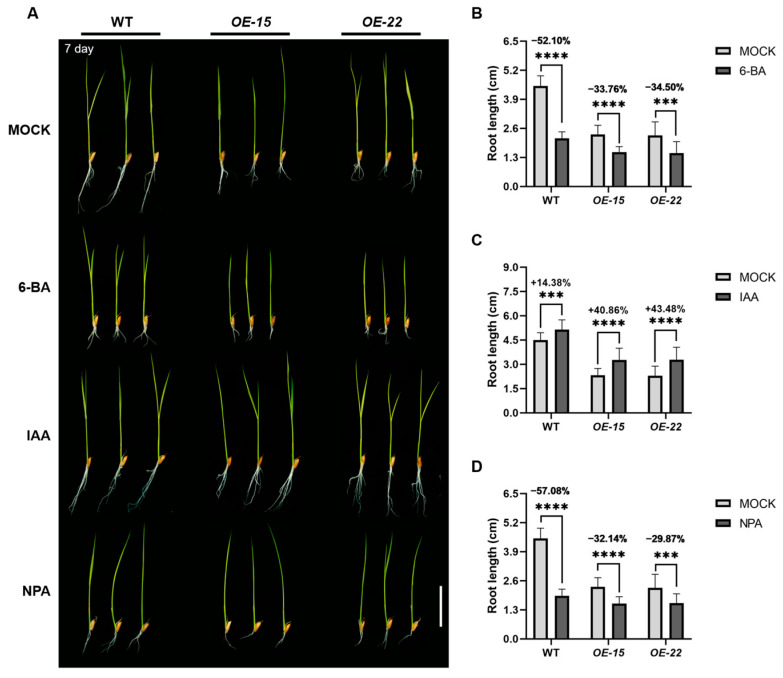
Hormone treatment. (**A**) Root phenotypes with or without hormone for 7 days; hormones involved include 6-BA, IAA, and NPA. Scale bar = 3 cm. (**B**) Root length statistics of seedlings treated with 6-BA, *n* ≥ 15. (**C**) Root length statistics of seedlings treated with IAA, *n* ≥ 22. (**D**) Root length statistics of seedlings treated with NPA, *n* ≥ 17. *** *p* < 0.001, **** *p* < 0.0001, determined by Student’s *t*-test.

As a type-B cytokinin response factor, the overexpression of *ORR3* significantly reduces the number of cells in the root meristematic zone, potentially due to aberrant cytokinin signaling. To test this hypothesis, we treated the WT and *ORR3-OE* lines with 6-benzylaminopurine (6-BA: cytokinin) and analyzed the plant height and the root length. After 7 days of growth in a medium containing 1 μM 6-BA, the root length of the WT plants treated with 6-BA decreased by about 52% compared to that of the control, mimicking the phenotype observed in the *ORR3-OE* lines. Moreover, the roots of *OE-15* and *OE-22* exhibited significantly enhanced resistance to 6-BA, with root length reductions of about 34% and 35%, respectively. Although plant height also decreased significantly in these lines, there was no significant difference in the extent of reduction (Figure 3A,B and Figure S3A). This suggests that *ORR3* overexpression inhibits root elongation primarily by suppressing cell division in the root tip.

Auxin has been shown to play a crucial role in plant root development [11]. Type-B cytokinin response factors regulate plant development by modulating cytokinin signaling. Cytokinins can inhibit cell division in the root tip meristem by suppressing auxin synthesis and distribution [36], thereby affecting the number of cells in the meristematic zone. Since the primary roots of monocotyledonous rice cease growth approximately 10 days after germination [6], we selected 7-day-old rice seedlings for a more systematic analysis. We cultivated the WT and *ORR3-OE* lines in media containing 1 μM indole-3-acetic acid (IAA) and 1 μM N-1-naphthylphthalamic acid (NPA: an inhibitor of auxin polar transport) for 7 days and analyzed the plant height and the root length. The root length exhibited significant differences, while the plant height remained largely unchanged (Figure 3B,C). The IAA treatment significantly increased the root length in both the WT and *ORR3-OE* lines, with increases of about 14% in the WT, about 41% in *OE-15*, and about 43% in *OE-22* (Figure 3A,C). This suggests that the IAA treatment partially restored the root phenotype of the *ORR3-OE* lines, implying that the auxin content in the root tips of *ORR3-OE* lines may be reduced. In contrast, NPA treatment significantly decreased the root length in both the WT and *ORR3-OE* lines, with reductions of about 57% in the WT, about 32% in *OE-15*, and about 30% in *OE-22* (Figure 3A,D). This indicates that *ORR3* overexpression conferred enhanced resistance to NPA in the rice seedling roots. These results suggest that the inhibition of root tip cell division by *ORR3* overexpression is closely related to the auxin metabolic pathway.

**Figure 4:**
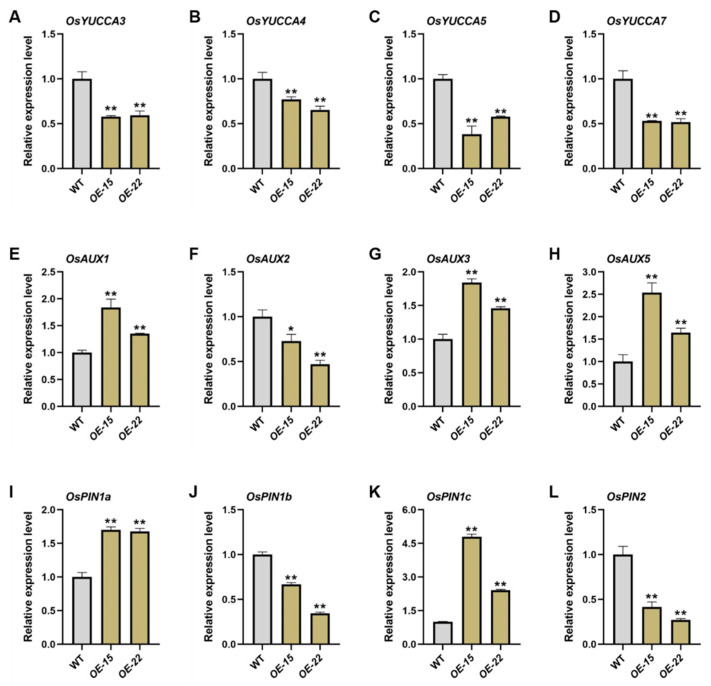
qRT-PCR analysis. (**A**–**D**) Expression of *OsYUCCA* gene in root tips of 7-day-old WT and *ORR3-OE* lines. (**E**–**H**) Expression of *OsAUX* gene in root tips of 7-day-old WT and *ORR3-OE* lines. (**I**–**L**) Expression of *OsPIN* gene in root tips of 7-day-old WT and *ORR3-OE* lines. * *p* < 0.05, ** *p* < 0.01, determined by Student’s *t*-test.

To further investigate this, we extracted total RNA from the primary and adventitious roots of the 7-day-old WT and *ORR3-OE* lines and performed qRT-PCR analysis on auxin-related genes. The results showed that the expression of the auxin biosynthesis genes *OsYUCCA3*, *OsYUCCA4*, *OsYUCCA5*, and *OsYUCCA7* was significantly downregulated in the root tips of *OE-15* and *OE-22* (Figure 4A–D). Combined with the finding that the IAA treatment partially restored the phenotype of the *ORR3-OE* lines (Figure 3A,C), this suggests that the reduced number of cells in the root tip meristematic zone of the *ORR3-OE* lines is associated with impaired auxin synthesis. Additionally, the transcription levels of the AUX family genes and the PIN family genes related to auxin polar transport, such as *OsAUX1*, *OsAUX3*, *OsAUX5*, *OsPIN1a*, and *OsPIN1c*, were upregulated in the root tips of *OE-15* and *OE-22*, while the transcription levels of *OsAUX2*, *OsPIN1b*, and *OsPIN2* were downregulated, all reaching significant or highly significant levels (Figure 4E–L). This correlates with the altered resistance of *ORR3-OE* lines to polar transport inhibitors (Figure 3A,D) and suggests that auxin polar transport is disrupted in the root tips of the *ORR3-OE* lines.

In summary, the overexpression of *ORR3* affects cytokinin signaling and inhibits root tip cell division by altering the auxin synthesis and transport pathways. This leads to a reduced number of cells in the root meristematic zone and restricts root growth.

### 2.4. The Overexpression of ORR3 Impacts Both Hormone Metabolism and Cell Wall Metabolism in the Root Tip

**Figure 5:**
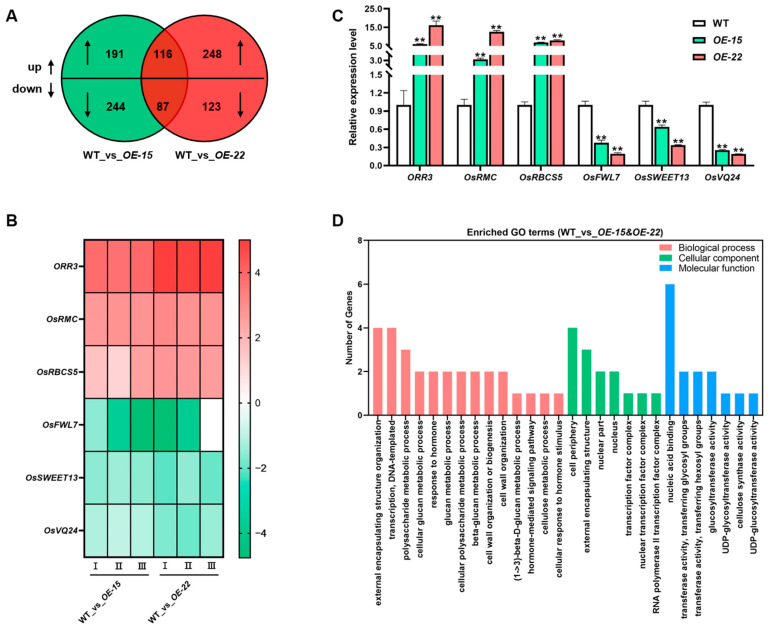
Transcriptome analysis. (**A**) DEGs Venn diagram. WT_vs_*OE-15* (green circle), WT_vs_*OE-22* (red circle), and WT_vs_*OE-15&OE-22* (overlapping region). Numbers indicate count of regulated genes. Up (upward-pointing arrow): up-regulated genes; down (downward-pointing arrow): down-regulated genes. (**B**) Heatmap of expression changes for randomly selected differential genes in transcriptome. Data displayed are derived from 3 biological replicates labeled as I, II, and III. (**C**) Expression analysis of randomly selected genes to validate accuracy of transcriptome. (**D**) Enriched gene ontology (GO) pathways among differentially expressed genes (DEGs). Enriched molecular function terms of DEGs between WT and *ORR3-OE* lines. ** *p* < 0.01, determined by Student’s *t*-test.

To further elucidate the function of *ORR3* in the root meristematic zone of young rice seedlings, we performed high-throughput RNA-seq on a pooled sample of the root tips of primary and adventitious roots from the 7-day-old WT, *OE-15*, and *OE-22* lines. The reliability of the transcriptomic data was validated through qRT-PCR (Figure 5B,C). Differentially expressed genes (DEGs) were identified based on the criteria of *p* ≤ 0.05 and a log2 fold-change ≥1, with the WT serving as the control for both the *OE-15* and *OE-22* lines. Analysis revealed that 116 DEGs were commonly upregulated, while 87 DEGs were commonly downregulated (Figure 5A).

Gene ontology (GO) term enrichment analysis indicated that for WT_vs_*OE-15&OE-22*, the 203 DEGs were mainly enriched in 138 pathways related to biological processes (BPs). Among these, 14 BPs were closely associated with cell wall metabolism, hormone signaling, and DNA transcription, such as external encapsulating structure organization (GO:0045229), transcription, DNA templates (GO:0006351), the *β*-glucan metabolic process (GO:0051273), cell wall organization (GO:0071555), the (1->3)- *β*-D-glucan metabolic process (GO:0006074), the hormone-mediated signaling pathway (GO:0009755), and the cellulose metabolic process (GO:0030243). Additionally, thirty-seven pathways related to cellular components (CCs) were enriched, with seven CCs associated with cell wall and nuclear transcription factors, such as the cell periphery (GO:0071944), the external encapsulating structure (GO:0030312), and the nuclear transcription factor complex (GO:0044798). Finally, sixty-one pathways related to molecular functions (MFs) were enriched, with seven MFs related to cell wall and DNA binding, such as nucleic acid binding (GO:0003676), glucosyltransferase activity (GO:0046527), UDP-glycosyltransferase activity (GO:0008194), cellulose synthase activity (GO:0016759), and UDP-glucosyltransferase activity (GO:0035251) (Figure 5D).

The GO pathway analysis suggests that the overexpression of *ORR3* affects the hormone signaling pathways and the cell wall metabolism pathways in the root tip, thereby regulating the development of young rice roots.

### 2.5. ORR3 Functions as a Transcriptional Activator That Regulates the Transcription of Cell Wall Metabolism-Related Genes

**Figure 6:**
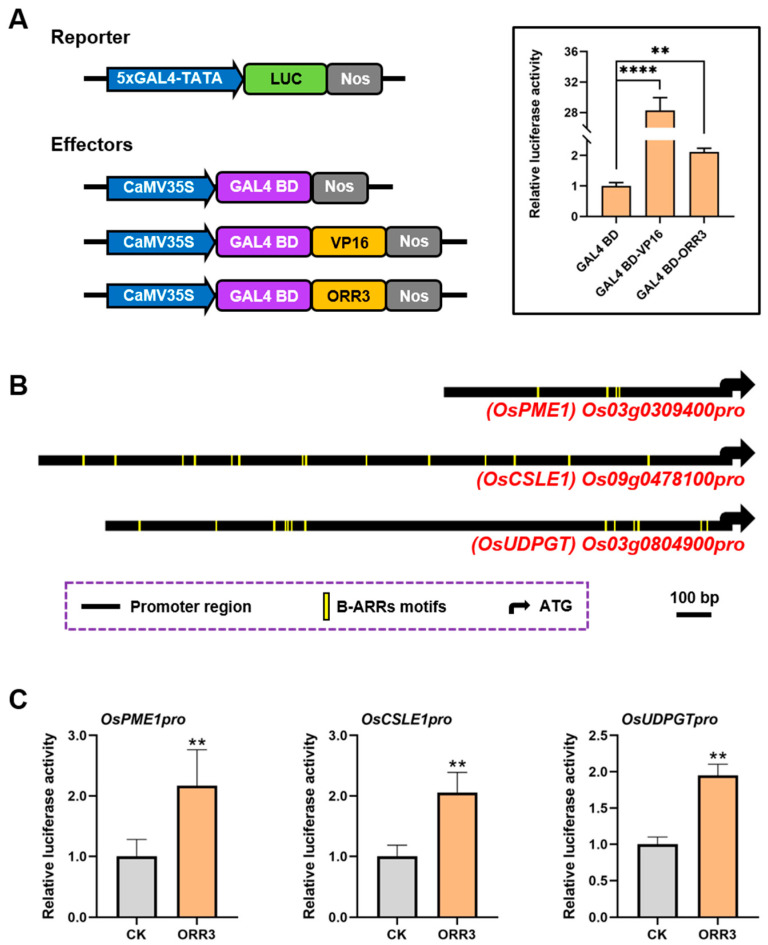
ORR3 directly binds to promoters of genes associated with cell wall metabolism. (**A**) Analysis of transcriptional activation of ORR3 using dual luciferase reporter assay system. Transactivation activity in rice protoplasts transfected with pUAS-fLUC reporter construct, effector constructs fused with GAL4BD, and p35S-rLUC normalization construct. (**B**) Schematic diagram depicting proximal promoter region is presented; binding site for B-ARR is represented by yellow vertical line. (**C**) Assessment of transcriptional activity of ORR3 protein on *OsPME1*, *OsCSLE1,* and *OsUDPGT* promoters using dual-luciferase reporter assay in rice protoplasts. ** *p* < 0.01, **** *p* < 0.0001, determined by Student’s *t*-test.

B-type cytokinin response factors have been reported to encode the GARP DNA-binding domain and function as transcription factors (Figure S1A) [32,33,34]. To determine the transcriptional activity of ORR3, we constructed a fusion expression vector of ORR3-GAL4 BD-LUC. GAL4 BD-LUC was used as a negative control, and VP16-GAL4 BD-LUC was used as a positive control [37]. These constructs were co-transformed into rice protoplasts. The results indicated that ORR3 acts as a transcriptional activator (Figure 6A).

Abnormal cell wall metabolism can affect the structure and function of the cell wall, thereby altering cell morphology or size [38,39]. In the overexpression lines of ORR3, significant changes in the root tip cell size were observed (Figure 2D,F), along with the differential expression of cell wall metabolism pathways (Figure S4). These findings collectively suggest that ORR3, as a transcriptional activator, may regulate the cell wall metabolism pathways. To test this hypothesis, we first analyzed the promoter sequences of cell wall metabolism-related genes that were commonly upregulated in the transcriptome data of *OE-15* and *OE-22*. We found that these promoters contained varying numbers of B-ARR binding elements [40,41] (Figure 6B).

To further investigate, we employed a dual-luciferase reporter system to assess LUC activity in rice protoplasts. Firefly LUC was driven by the promoters of candidate genes, while Renilla (REN) LUC was driven by the CaMV35S promoter. The only difference between the reporter and effector plasmids was the inclusion of the ORR3 protein. After transforming these plasmids into rice protoplasts, we measured LUC activity. The results showed that ORR3 could directly bind to the promoters of the pectin methylesterase-encoding gene *OsPME1*, the cellulose synthase-encoding gene *OsCSLE1*, and the pectin synthesis-related gene *OsUDPGT*, thereby activating their transcription (Figure 6C). These findings indicate that ORR3, as a transcriptional activator, directly activates the transcription of the cell wall metabolism-related genes in rice root tips. This mechanism may underlie the changes in cell size in the root meristematic zone observed in *ORR3* overexpression lines.

### 2.6. The Overexpression of ORR3 Affects Rice Grain Development

**Figure 7:**
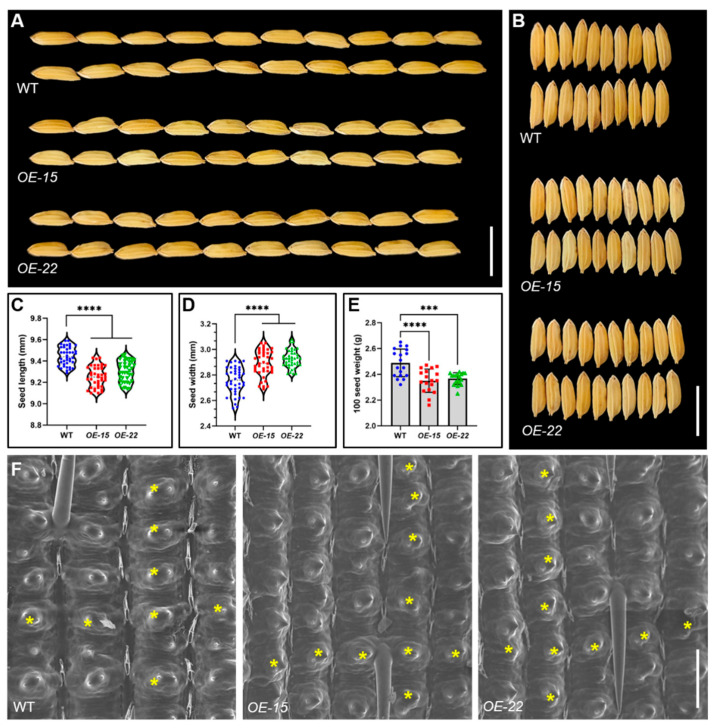
Overexpression of *ORR3* affects rice grain development. (**A**) Grain length phenotypes of WT and *ORR3-OE* lines. Scale bar = 1 cm. (**B**) Grain width phenotypes of WT and *ORR3-OE* lines. Scale bar = 1 cm. (**C**) Statistics of grain length, *n* = 40. (**D**) Statistics of grain width, *n* = 40. (**E**) Statistics of 100-grain weight, *n* = 18. (**F**) Scanning electron micrographs of outer epidermal cells of hulls. Scale bar = 100 um. Yellow asterisks denote cell count. *** *p* < 0.001, **** *p* < 0.0001, determined by Student’s *t*-test.

We observed the WT and *ORR3-OE* plants in the field and found that there was no significant difference in plant height between *OE-15* and *OE-22* and the WT during the tillering and maturity stages (Figure S5A,B). Further measurements of the grains revealed that compared to the WT, the grain length of *OE-15* and *OE-22* was significantly reduced (Figure 7A,C), while the grain width was significantly increased (Figure 7B,D), ultimately leading to a significant decrease in the weight of 100 grains (Figure 7E). These results suggest that the overexpression of *ORR3* may have a negative impact on rice grain development. To investigate the cellular differences, we examined the outer epidermal cells of the hulls from both the WT and *ORR3-OE* plants using scanning electron microscopy. The results showed that the number of epidermal cells per unit area in the longitudinal and transverse directions of the *ORR3-OE* hulls increased compared to that of the WT, indicating that the cells had become smaller (Figure 7F). Additionally, observations using a stereomicroscope and scanning electron microscopy revealed no significant morphological differences in the anthers and the pistils of the *ORR3-OE* plants compared to those of the WT (Figure S5C–E). However, a closer examination of the anther epidermal cells using scanning electron microscopy showed that the overexpression of *ORR3* led to a significant reduction in the structure of the cuticle layer, with clearer cell outlines and smaller cell sizes (Figure S5F). These findings collectively indicate that *ORR3* is involved in the regulation of rice grain development.

## 3. Discussion

### 3.1. Overexpression of ORR3 Negatively Regulates the Development of Young Rice Roots

Some B-type cytokinin response regulators are involved in the regulation of rice growth and development. For example, *OsRR21* participates in rice panicle development [41], *OsRR22* mutants show significantly enhanced salt tolerance [42], and *OsRR24* is involved in regulating rice fertility [43,44]. *OsRR30* is crucial for rice heading and yield [45,46]. The B-type cytokinin response factors play crucial roles not only in shoot development, but also in the regulation of plant root growth and development. For instance, Ma et al. [47] reported that deletion of B-type ARR led to elongated roots in plants. Worthen et al. [44] found that the primary roots of triple rice mutants *OsRR21*, *OsRR22*, and *OsRR23* were significantly shorter compared to the WT. In *Arabidopsis thaliana*, the primary root of the *arr1* mutant was longer than that of the WT [48,49]. Similarly, the roots of *arr1/10/12* triple mutants were longer than those of the WT [50]. However, due to differences in the mutation sites, Yokoyama et al. [51] and Argyros et al. [52] reported contrasting results, with the *arr1/10/12* mutants exhibiting significantly shorter roots compared to those of the WT. Despite these findings, the studies on the regulation of young root development by B-type ARRs in rice are relatively limited. In this study, we constructed transgenic plants overexpressing *ORR3*. These plants exhibited significantly shorter primary and adventitious roots, as well as reduced meristematic zone lengths compared to those of the WT. This suggests that the overexpression of *ORR3* negatively regulates the development of young rice roots. Further analysis revealed that this effect was likely due to a reduction in both the number and length of cells in the root meristematic zone.

### 3.2. ORR3 May Regulate the Number of Longitudinal Cells in the Root Meristematic Zone by Modulating Auxin Levels or Signaling

Auxin plays a crucial role in root development in plants. *OsMADS25* regulates rice root growth and development through the Aux/IAA-mediated auxin signaling pathway [16]. The root apical meristem (RAM) exhibits a high capacity for IAA synthesis, which is transported upwards by specific proteins, such as AUX and PIN [53,54]. Additionally, there is interaction between auxin and cytokinin. Cytokinin inhibits cell division in the RAM by affecting auxin synthesis and transport [36]. A higher endogenous ratio of IAA to cytokinin (CTK) is conducive to adventitious root formation, while a lower ratio is unfavorable for root induction [19]. The IAA treatment partially restored the root phenotype of *ORR3-OE*, suggesting that auxin synthesis or polar transport may be impaired in the root tips of *ORR3-OE*. Further investigation revealed that the expression of auxin synthesis genes, YUCCAs, was significantly downregulated in *OE-15* and *OE-22* (Figure 4A–D), indicating that *ORR3* may reduce auxin synthesis by downregulating the transcription of *OsYUCCAs*. The treatment with the auxin polar transport inhibitor NPA showed increased resistance in *ORR3-OE*, suggesting that auxin polar transport is disrupted in the root tips of *ORR3-OE*. Moreover, the transcription levels of auxin transport genes *OsPINs* and *OsAUXs* were significantly altered in *OE-15* and *OE-22* (Figure 4E–L). Therefore, the rice type-B cytokinin response regulator *ORR3* may play a key role in the cytokinin-mediated regulation of auxin synthesis and transport in the root tip, thereby inhibiting cell division and significantly reducing the number of cells in the root meristematic zone. Our study provides new insights into how cytokinin regulates the longitudinal cell number in the root meristematic zone through auxin.

### 3.3. ORR3 May Regulate the Length of Root Meristematic Zone Cells by Affecting Cell Wall Metabolism

Plant cells possess a complex cell wall primarily composed of polysaccharide polymers, including cellulose, hemicellulose, and pectin [55]. Numerous studies have reported that the genes involved in cell wall metabolism can influence rice root development by affecting cell morphology. For example, the rice gene *OsGLU1* encodes a membrane-bound endo-1,4-*β*-D-glucanase. In its mutant, the cellulose content in the roots is reduced, while the pectin content increases and root cell elongation is impeded [56]. Additionally, the rice cellulose synthase gene *SUS1* promotes root cell elongation [57]. The transcriptomic data from root tips suggest that *ORR3* may be involved in regulating rice young root development through cell wall metabolism pathways. Analysis revealed that the upregulation of the pectin methylesterase-encoding gene *OsPME1* may lead to a decrease in pectin [58], while the upregulation of the UDP-rhamnose rhamnosyltransferase-encoding gene *OsUDPGT* may result in an increased pectin content [59]. The upregulation of the brown planthopper resistance gene *OsBph40* may lead to increased cellulose content [60], and the downregulation of the xylanase-encoding gene *OsXyn1* may cause a reduction in cellulose content [61]. The upregulation of the xylanase inhibitor-encoding gene *Os01g0937000* may lead to an increased hemicellulose content [62], while the downregulation of the xylan arabinose 2-O-xylosyltransferase-encoding gene *OsXAXT1* may result in decreased hemicellulose content [63]. Collectively, these findings indicate that the overexpression of *ORR3* may lead to disordered cell wall metabolism in root tip cells.

Cellulose and pectin are major components of the cell wall, providing important tensile strength to the wall matrix [64]. Pectin methylesterase (PME) plays a crucial role in pectin metabolism within the plant cell wall. PME catalyzes the de-esterification of pectin, thereby increasing cell wall rigidity and reducing cell extensibility [65]. In rice, the overexpression of *OsPME14* under Al-induced conditions significantly inhibits root elongation compared to that of the WT [66]. Cellulose, through its highly crystalline structure, the interweaving of microfibrils, and its synergistic interactions with other components, imparts a rigid characteristic to the cell wall. Hemicellulose can also enhance the overall strength of the cell wall by cross-linking with cellulose [67]. The cellulose synthase-like (CSL) gene family is involved in the synthesis of cellulose and hemicellulose in plants. For example, mutations in the rice *OsCslF6* gene result in a significant reduction in cellulose content, indicating its close association with cellulose synthesis [68]. Similarly, the CSLE gene likely plays a crucial role in regulating cell shape and size, while providing essential mechanical support during plant growth and development. In this study, we found that ORR3 possesses transcriptional activation activity. It can directly activate the transcription of *OsPME1*, *OsCSLE1*, and *OsUDPGT*, thereby regulating cell wall metabolism in rice root tip cells. This regulation may subsequently limit cell length by influencing the structure and function of the cell wall.

## 4. Materials and Methods

### 4.1. Construction of Plant Materials and Phenotypic Observation

In this study, Jinhui 10 (J10; *Oryza sativa* ssp. *indica*) was used as the wild-type (WT) rice material. To generate overexpression transgenic lines of the *ORR3* gene, the full-length coding sequence (CDS) of *ORR3* was amplified from the root cDNA of J10 and cloned into the binary vector pTCK303 under the control of the ubiquitin promoter. The recombinant plasmids were introduced into J10 via Agrobacterium tumefaciens-mediated transformation. The primers used for cloning are listed in Table S1. For phenotypic observation, the plant materials were cultivated in half-strength Murashige and Skoog (1/2 MS) culture solution. Growth was conducted in a light incubator under a 12 h light/12 h dark photoperiod at a temperature of 30 °C for a duration of 5–10 days. Light intensity was maintained at 300 µmol photons m^−2^ s^−1^ (white light), and relative humidity was kept at 60%. The lengths of both the primary root and adventitious roots were measured, and photographs were taken to document the phenotypic differences. All the experimental materials were cultivated and reproduced at the Rice Research Institute at Southwest University, localized in Xiema Town, Beibei District, Chongqing, China.

### 4.2. RNA Isolation and qRT-PCR Analysis

Total RNA was isolated from the seedling root apicals of the WT and transgenic plants of *ORR3* using the RNAprep Pure Plant RNA Purification Kit (Tiangen, Beijing, China). First-strand cDNA was synthesized using the SuperScript III Reverse Transcriptase Kit (Invitrogen, Carlsbad, CA, USA). Quantitative reverse-transcription polymerase chain reaction (qRT-PCR) analysis was performed using the strategy described previously [69], and actin was used as the endogenous control. At least three replicates were performed, from which the mean value was used to represent the expression level. The primers used for cloning are listed in Table S1 [9,70,71,72,73,74,75,76,77].

### 4.3. In Situ Hybridization

For the *ORR3* probe, its cDNA was amplified and labeled using the DIG RNA Labeling Kit (Roche, Basel, Switzerland). The pretreatment of sections, hybridization, and immunological detection were performed as described previously [69,78]. The primers used are listed in Table S1.

### 4.4. Subcellular Localization

The full-length coding region of *ORR3* was amplified and cloned into the expression cassette *CaMV35S*-GFP-NOS (pAN580) to generate ORR3-GFP fusion vectors. Then, *CaMV35S*-GFP and *CaMV35S*-ORR3-GFP plasmids were transformed into rice protoplasts as previously described [69]. After overnight incubation at 28 °C, GFP fluorescence was observed with a confocal laser scanning microscope (Olympus, Tokyo, Japan). For subcellular localization in *N. benthamiana*, the *GV3101* strain of Agrobacterium tumefaciens harboring *UBQ10*-GFP and *UBQ10*-ORR3-GFP plasmids was cultured; following this, the *N. benthamiana* leaves were infiltrated and maintained in darkness for 48–72 as previously described [79,80]. The primers used are listed in Table S1.

### 4.5. Phylogenetic Analysis

The full-length amino acid sequence of rice ORR3 was retrieved from the National Center for Biotechnology Information (NCBI) database (https://www.ncbi.nlm.nih.gov/) (accessed on 23 September 2024). Homologous genes in both the monocotyledonous and dicotyledonous plants were identified using the Phytozome database (https://phytozome-next.jgi.doe.gov/) (accessed on 21 November 2024) as per the methodology described by Goodstein et al. [81]. Multiple sequence alignments were conducted using MEGA 7 software, and phylogenetic trees were constructed with the same software employing the maximum likelihood method and bootstrap analysis as recommended by Kumar et al. [82]. Subsequently, the trees were refined using ITOL (https://itol.embl.de) (accessed on 25 November 2024) following the guidelines provided by Letunic and Bork [83].

### 4.6. Microscopy Analysis

Root apicals from the WT and transgenic plants of *ORR3* were collected and fixed in formalin–acetic–alcohol (FAA) solution (50% ethanol, 0.9 M glacial acetic acid, and 3.7% formaldehyde) overnight at 4 °C, dehydrated in a series of ethanol solutions, substituted with xylene, and embedded in paraffin (Sigma, St. Louis, MO, USA). The samples were sectioned to 8 μm, transferred onto poly-l-Lys-coated glass slides, deparaffinised with xylene, and dehydrated through a series of ethanol solutions. The sections were stained with 1% Fast Green (Amresco, Framingham, MA, USA), dehydrated through a series of ethanol solutions, infiltrated with xylene, and mounted under a coverslip. Light microscopy was performed using the Eclipse E600 microscope (Nikon, Tokyo, Japan) [84]. To observe tissue length in the root a meristematic zone of rice, the root apicals were stained with 1% acetate-magenta staining solution for 10 min, and then rinsed with 45% acetic acid and photographed using an Eclipse E600 microscope (Nikon, Japan) [35].

### 4.7. Transcriptome Analysis

Total RNA was extracted from 1 cm length of the root apicals of the WT, *ORR3-OE-15* (*OE-15*), and *ORR3-OE-22* (*OE-22*), with three replicates per sample, respectively. The samples were sequenced using the DNBSEQT7 genetic sequencer at Tsingke Biotech Co., Ltd. (Beijing, China). The raw data were mapped onto the rice reference genome using HISAT2 software with default parameters [85]. Gene expression levels were quantified using StringTie and expressed as fragments per kilobase of transcript per million mapped reads (FPKMs) [86]. Differentially expressed genes (DEGs) were identified considered *p* ≤ 0.05 and a log2 fold-change ≥ 1. Gene ontology (GO) enrichment analysis was performed to identify the significantly enriched biological processes in *ORR3-OE*. The RNA sequencing data were deposited in the NCBI Sequence Read Archive (SRA) under accession PRJNA1231205.

### 4.8. Hormone Treatments

In order to determine the responses of the *ORR3-OE* plants to the different hormones, we treated the WT, *OE-15*, and *OE-22* plants with culture solutions containing 1 μM 6-benzylaminopurine (6-BA) (S18017, Yuanye, Shanghai, China), 1 μM indole-3-acetic acid (IAA) (C16431, Coolaber, Beijing, China), and 1 μM N-1-naphthylphthalamic acid (NPA) (CN7710, Coolaber, Beijing, China), respectively, for 7 days. We then photographed the plants and measured their root lengths.

### 4.9. Analysis of Transcriptional Activity and Transient Expression Assay

The transcriptional activity of full-length ORR3 proteins was analyzed in rice protoplasts using the dual luciferase reporter assay system; VP16, a transcriptional activator, was used as the positive control, and GAL4-BD was regarded as the negative control. The promoter regions of the target gene translation initiation codon were cloned into the pGreenII0800-LUC vector. The CDS of full-length ORR3 proteins were under the control of the CaMV35S promoter. Different combinations of plasmids were transformed into rice protoplasts. The relative activities of LUC/REN were measured using a GloMax 20/20 luminometer (Promega, Madison, WI, USA). The experimental data reflect the average of three technical replicates, with the experiment being repeated three times [80,87,88]. The primers used are listed in Table S1.

### 4.10. Statistical Analyses

Comparisons were made by two-tailed Student’s *t*-test or one-way ANOVA and the Duncan multiple comparisons test (*p* < 0.05) using statistical software GraphPad Prism 9.5.0 [89]. The asterisk (*) symbols indicate significant differences relative to the wild type at * *p* < 0.05, ** *p* < 0.01, *** *p* < 0.001, **** *p* < 0.0001.

## 5. Conclusions

The development of young roots is essential for high yield in cereal crops [1,2]. Therefore, it is crucial to investigate the mechanisms underlying the development of primary and adventitious roots during the seedling stage. In this study, we explored the function of the type-B cytokinin response factor *ORR3* and found that it likely regulates the number and size of cells in the root meristematic zone through two pathways. First, it responds to cytokinin signaling to modulate auxin synthesis and transport. Second, it acts as a transcriptional activator to influence cell wall metabolism. Both the pathways negatively regulate the growth of young rice roots. Moreover, agronomic trait analysis revealed that *ORR3* may also negatively regulate rice grain development, suggests that *ORR3* could be a potential target for improving rice productivity. In summary, our findings enrich the molecular regulatory network of crop root development and provide new targets and ideas for the genetic improvement of crops.

## Data Availability

Data are contained within the article and Supplementary Materials.

## Supplementary Materials

The following supporting information can be downloaded at: https://www.mdpi.com/article/10.3390/plants14111627/s1, Figure S1: ORR3 protein analysis; Figure S2: Overexpression of *ORR3* inhibits rice root development; Figure S3: Hormone treatment; Figure S4: Candidate cell wall metabolism-related genes regulated by ORR3; Figure S5: Phenotypic identification; Table S1: Primer sequences used in this study.

## Accession Numbers

*ORR3* (Os02g0796500), *OsPIN1a* (Os06g0232300), *OsPIN1b* (Os02g0743400), *OsPIN1c* (Os11g0137000), *OsPIN2* (Os06g0660200), *OsAUX1* (Os01g0856500), *OsAUX2* (Os03g0244600), *OsAUX3* (Os05g0447200), *OsAUX5* (Os11g0169200), *OsYUCCA3* (Os01g0732700), *OsYUCCA4* (Os01g0224700), *OsYUCCA5* (Os12g0512000), *OsRMC* (Os04g0659300), *OsRBCS5* (Os12g0291400), *OsFWL7* (Os03g0830500), *OsSWEET13* (Os12g0476200), *OsVQ24* (Os06g0618700), *OsPME1* (Os03g0309400), *OsCSLE1* (Os09g0478100), *OsBph40* (Os04g0166000), *OsXyn1* (Os03g0672900), *OsXAXT1* (Os06g0475400), *OsUDPGT* (Os03g0804900).
