# Supplementary material for: The Overexpression of ORR3 Negatively Regulates the Growth of Young Rice Roots by Reducing the Cell Size and the Number in the Root Meristematic Zone
Source: Plants (Basel). 2025 May 27;14(11):1627. doi: 10.3390/plants14111627 (PMC12157236; doi:10.3390/plants14111627)
Supplement: Supplementary file 1 [file plants-14-01627-s001.zip › plants-3623679-supplementary.pdf]

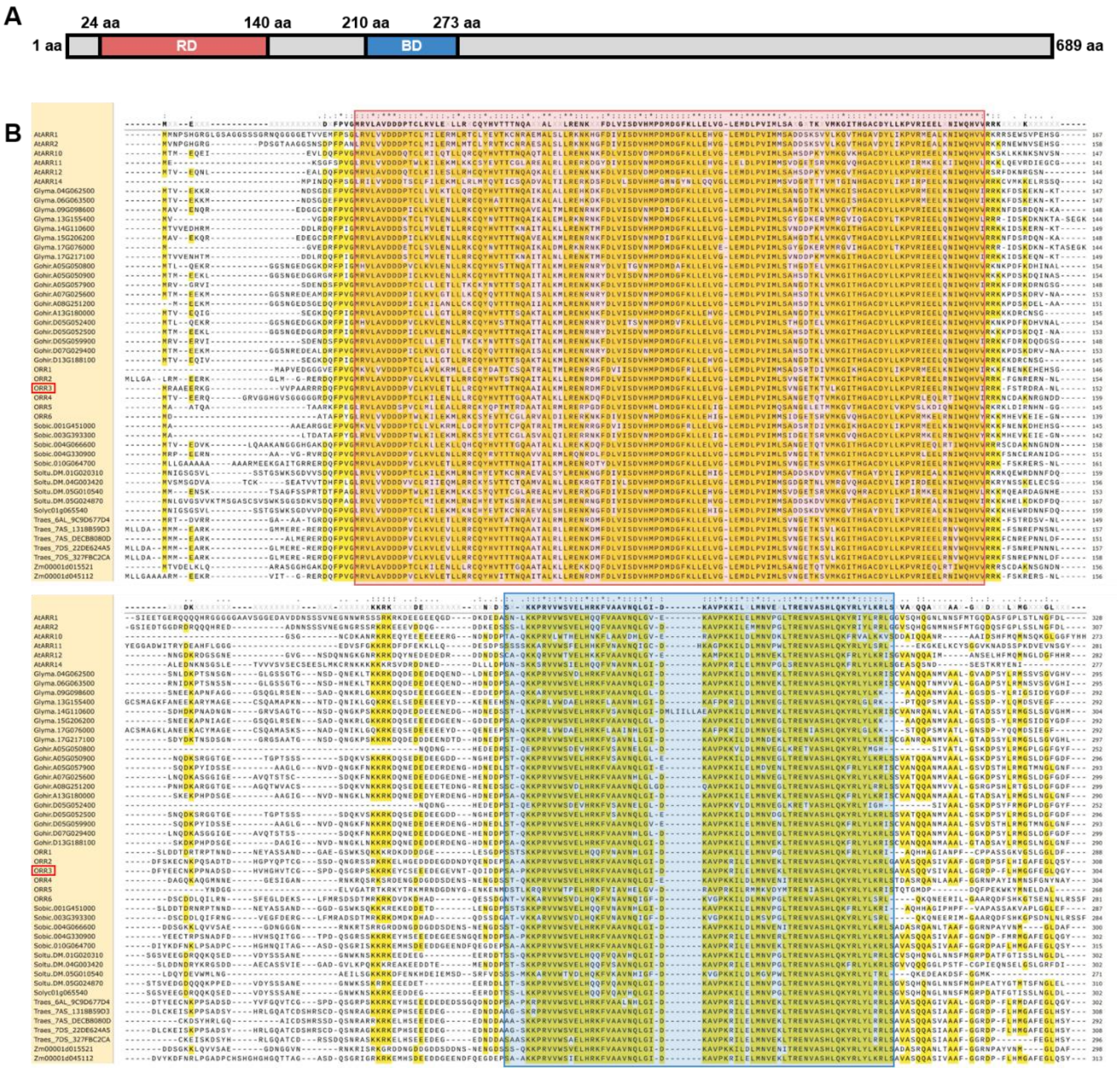

Figure S1. ORR3 protein analysis. (**A**) The structure of ORR3 protein, full-length 689 amino acids (aa), includes a response domain (RD) (red box, 24 aa to 140 aa) and a DNA-binding domain (BD) (blue box, 210 aa to 273 aa). (**B**) Protein sequence alignment, the red box corresponds to the RD domain, the blue box corresponds to the BD domain.

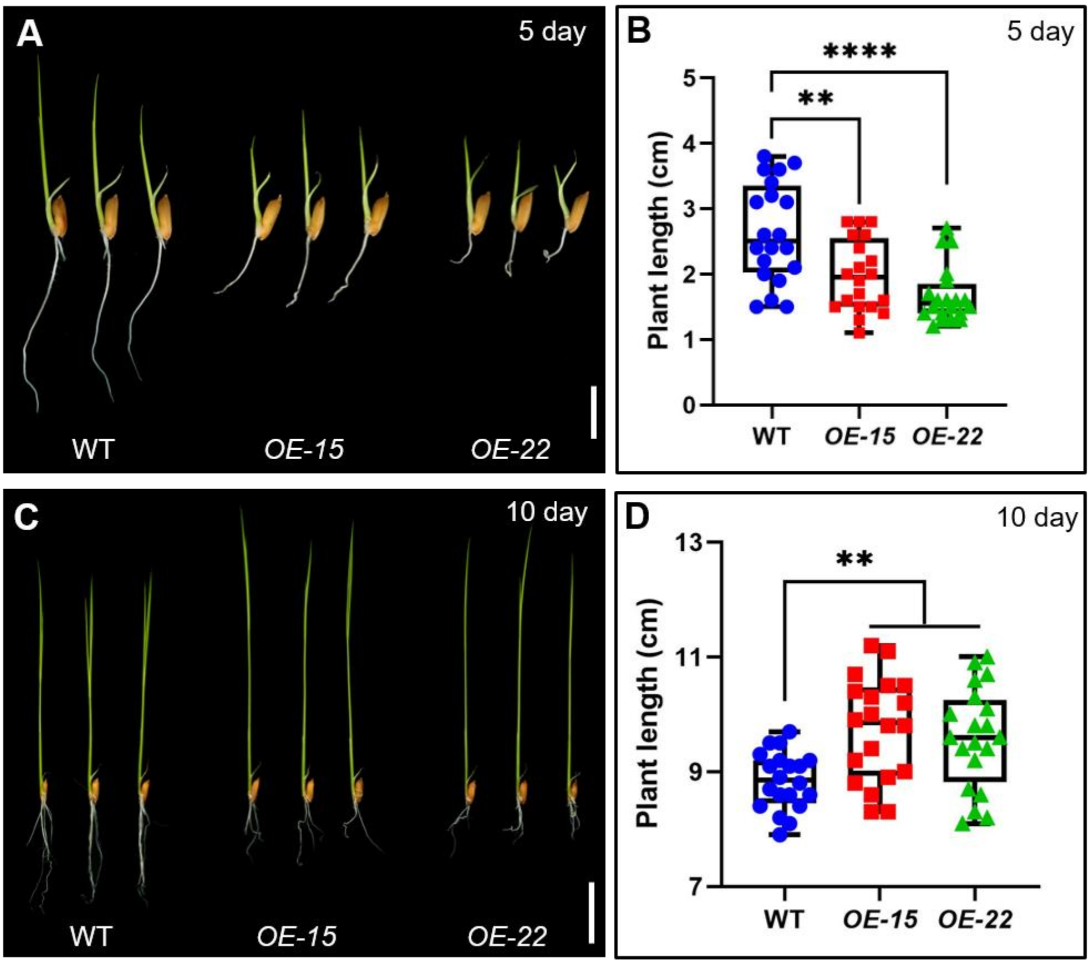

Figure S2. Overexpression of ORR3 inhibits rice root development. (**A**) 5-day-old seedlings, scale bar = 1 cm.(**B**) Plant height of 5-day-old seedlings, n = 20.(**C**) 10-day-old seedlings, scale bar = 3 cm. (**D**) Plant height of 10-day-old seedlings, n = 20. $^{**}p < 0.01$, $^{****}p < 0.0001$, determined by Student's t-test.

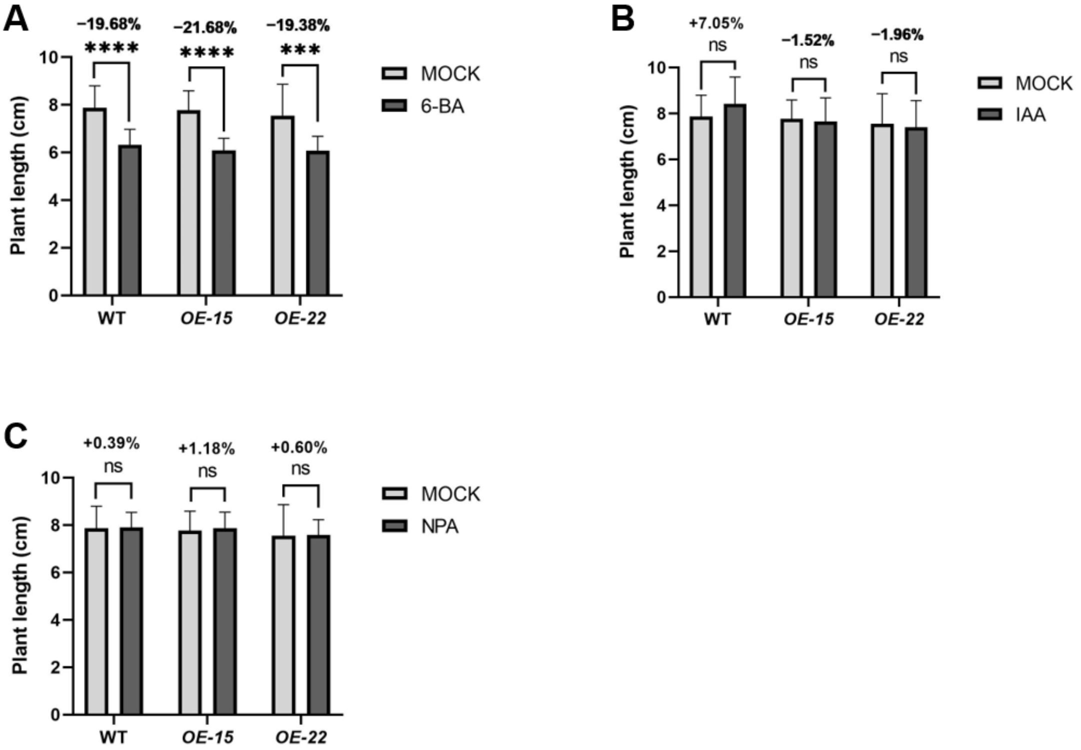

Figure S3. Hormone treatment. (A) Plant height statistics of seedlings treated with 6-BA, $n \geq 15$. (**B**) Plant height statistics of seedlings treated with IAA, $n \geq 22$. (**C**) Plant height statistics of seedlings treated with NPA, $n \geq 17$. ns: no significance, ***$p < 0.001$, ****$p < 0.0001$, determined by Student's t-test.

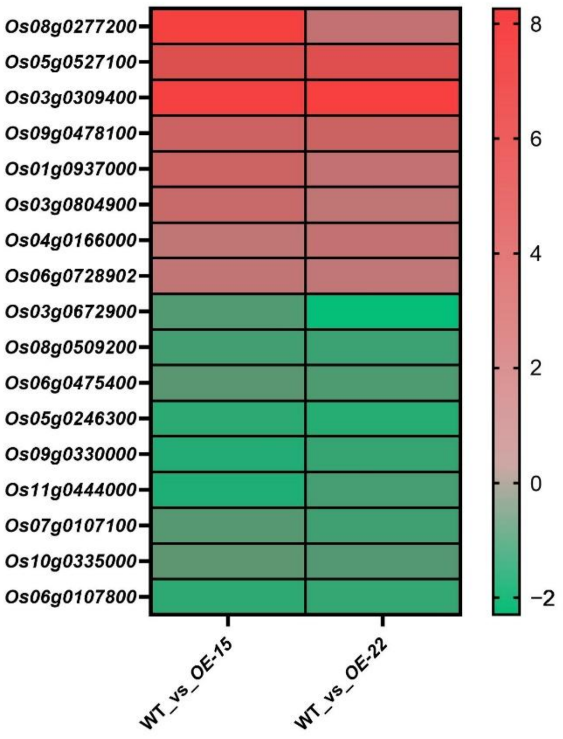

Figure S4. Candidate cell wall metabolism-related genes regulated by ORR3. WT_vs_*OE-15*: *ORR3-OE-15* lines compared with WT, WT_vs_*OE-22*: *ORR3-OE-22* lines compared with WT.

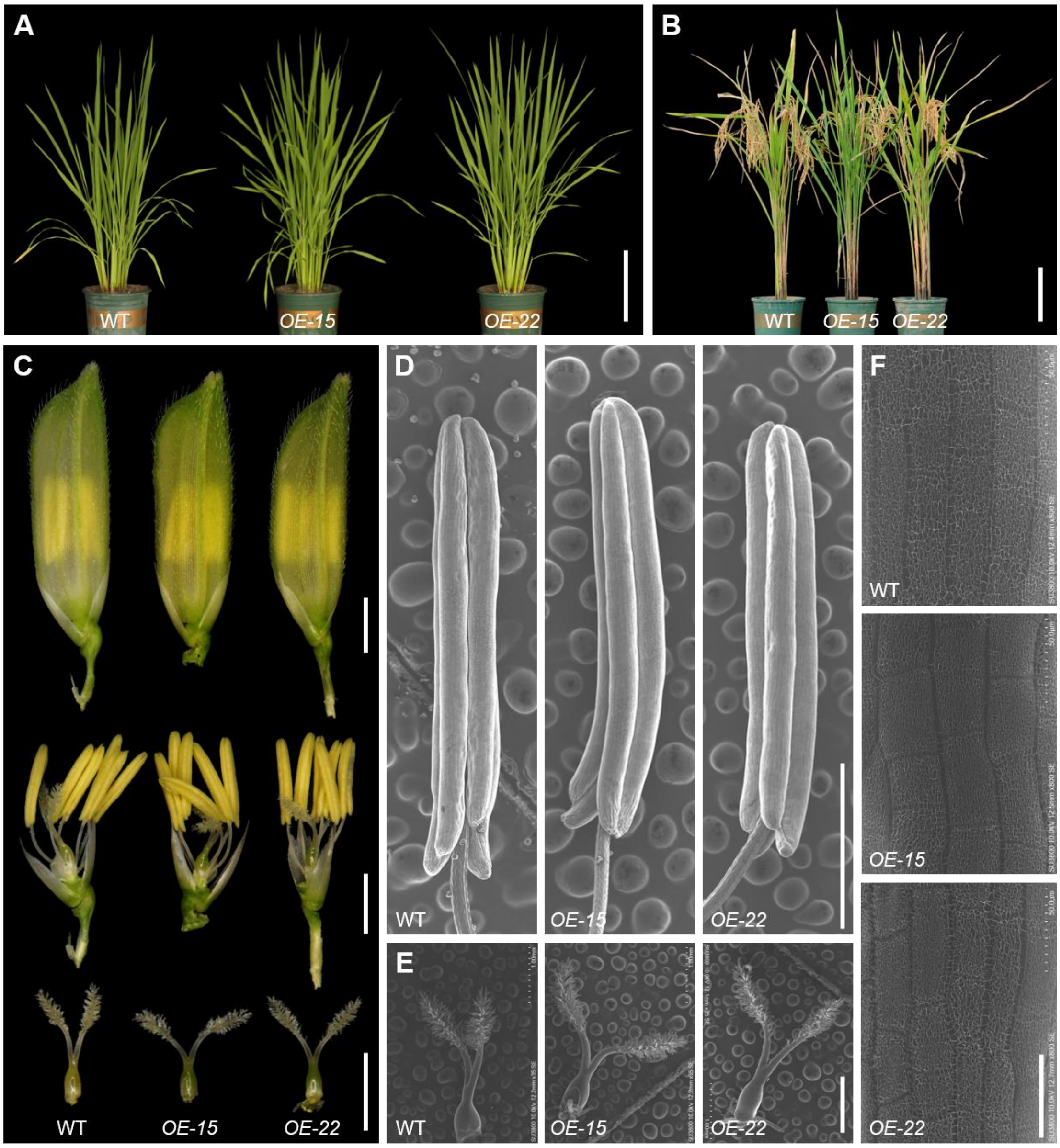

Figure S5. Phenotypic identification. (**A**) WT and *ORR3-OE* lines at tillering stage, scale bar = 20 cm. (**B**) WT and *ORR3-OE* lines at maturity, scale bar = 20 cm. (**C**) Morphological observation of reproductive organs, scale bar = 2 mm. (**D**) Scanning electron microscope of anther, scale bar = 1 mm. (**E**) Scanning electron microscope of pistil, scale bar = 1 mm. (**F**) Scanning electron microscope of anther epidermis, scale bar = 50 um.
